# Magnetoencephalography Responses to Unpredictable and Predictable Rare Somatosensory Stimuli in Healthy Adult Humans

**DOI:** 10.3389/fnhum.2021.641273

**Published:** 2021-04-14

**Authors:** Qianru Xu, Chaoxiong Ye, Jarmo A. Hämäläinen, Elisa M. Ruohonen, Xueqiao Li, Piia Astikainen

**Affiliations:** ^1^Institute of Brain and Psychological Sciences, Sichuan Normal University, Chengdu, China; ^2^Jyväskylä Centre for Interdisciplinary Brain Research, Department of Psychology, Faculty of Education and Psychology, University of Jyväskylä, Jyväskylä, Finland; ^3^Human Information Processing Laboratory, Psychology, Faculty of Social Sciences, Tampere University, Tampere, Finland

**Keywords:** deviance detection, magnetoencephalography, predictability, prediction error, somatosensory

## Abstract

Mismatch brain responses to unpredicted rare stimuli are suggested to be a neural indicator of prediction error, but this has rarely been studied in the somatosensory modality. Here, we investigated how the brain responds to unpredictable and predictable rare events. Magnetoencephalography responses were measured in adults frequently presented with somatosensory stimuli (FRE) that were occasionally replaced by two consecutively presented rare stimuli [unpredictable rare stimulus (UR) and predictable rare stimulus (PR); *p* = 0.1 for each]. The FRE and PR were electrical stimulations administered to either the little finger or the forefinger in a counterbalanced manner between the two conditions. The UR was a simultaneous electrical stimulation to both the forefinger and the little finger (for a smaller subgroup, the UR and FRE were counterbalanced for the stimulus properties). The grand-averaged responses were characterized by two main components: one at 30–100 ms (M55) and the other at 130–230 ms (M150) latency. Source-level analysis was conducted for the primary somatosensory cortex (SI) and the secondary somatosensory cortex (SII). The M55 responses were larger for the UR and PR than for the FRE in both the SI and the SII areas and were larger for the UR than for the PR. For M150, both investigated areas showed increased activity for the UR and the PR compared to the FRE. Interestingly, although the UR was larger in stimulus energy (stimulation of two fingers at the same time) and had a larger prediction error potential than the PR, the M150 responses to these two rare stimuli did not differ in source strength in either the SI or the SII area. The results suggest that M55, but not M150, can possibly be associated with prediction error signals. These findings highlight the need for disentangling prediction error and rareness-related effects in future studies investigating prediction error signals.

## Introduction

The ability to detect changes in the stimulus environment is crucial to an organism’s survival. Equally important is the capacity to learn contingencies between stimuli and to anticipate future events based on learned patterns in stimuli. Accurate predictions of future events can advance cognitive functioning related to perception and action in a fundamentally important manner (Bar, 2007).

According to the predictive coding theory (Friston, 2005), neural networks constantly learn the statistical regularities of the surrounding stimulus environment and make predictions of future events. When the input information does not match with the prediction, the lower sensory areas send a prediction error signal into the higher cortical areas (recent findings also extend this hierarchical pattern of predictive coding framework to subcortical structures, see Parras et al., 2017; Carbajal and Malmierca, 2018) and modify the prediction (Friston, 2005; Garrido et al., 2009; Stefanics et al., 2014). This new prediction is then sent backward to the lower areas, where it is again compared with the new sensory input signals.

In experimental research, an oddball stimulus condition, wherein a standard stimulus is rarely and randomly replaced by a deviant stimulus, is a feasible tool for studying predictive coding. An event-related potential, called mismatch negativity [MMN or MMNm when investigating with magnetoencephalography (MEG)] (Näätänen et al., 1978, 2010), is elicited by the deviant stimulus and is suggested to reflect prediction error (Friston, 2005; Garrido et al., 2009; Wacongne et al., 2012; Stefanics et al., 2014; Carbajal and Malmierca, 2018). MMN was originally found in the auditory modality (Näätänen et al., 1978) but was later reported as well for deviant stimuli in the visual (e.g., Stefanics et al., 2012; Astikainen et al., 2013; Xu et al., 2018; for reviews, see Czigler, 2007; Kimura et al., 2011; Stefanics et al., 2014; Kremláček et al., 2016), olfactory (e.g., Krauel et al., 1999; for a review, see Pause and Krauel, 2000), and somatosensory (e.g., Shinozaki et al., 1998; Spackman et al., 2007; Strömmer et al., 2014, 2017; for a review, see Näätänen, 2009) modalities.

Here, we focus on the somatosensory mismatch response [sMMR, instead of MMN due to its positive polarity in some previous electroencephalography (EEG) measurements], which is less studied than its auditory and visual counterparts. The sMMR has been observed for changes in stimulus location (Shinozaki et al., 1998; Huang et al., 2005; Restuccia et al., 2009; Strömmer et al., 2014, 2017; Yamashiro et al., 2014; Shen et al., 2018; Hautasaari et al., 2019; for animal models, see: Astikainen et al., 2001; Musall et al., 2017), duration (Akatsuka et al., 2005; Spackman et al., 2007, 2010; Zhao et al., 2014), intensity (Mima et al., 1998; Ostwald et al., 2012), frequency (Kekoni et al., 1997; Spackman et al., 2007), and omissions of the stimuli (Tesche and Karhu, 2000; Naeije et al., 2018). However, one critical confounder should be considered in the context of all the previously mentioned studies, namely, that the probability of the rare stimulus in the traditional oddball paradigm is always smaller than the probability of the standard stimulus and that probability, as such, affects the brain responses (Hari et al., 1990). One possible neural mechanism underlying probability effects is neural adaptation (May et al., 1999; May and Tiitinen, 2010), in which the neural populations responding to frequently presented standard stimuli can be more adapted than those responding to the rare deviant stimuli. Therefore, larger responses can be elicited for deviant stimuli than for standard stimuli (May and Tiitinen, 2010).

For auditory and, to some extent, for visual experiments as well, several different control conditions have been developed to control for possible adaptation effects for MMN elicitation. The many-standards condition (also called the equal-probability condition) is currently the most frequently used (Schröger and Wolff, 1996; Jacobsen and Schröger, 2001). In human auditory oddball studies, the results from the many-standards control condition suggest that the differential responses found in the oddball paradigm (MMN) may not be explained by adaptation alone (Jacobsen and Schröger, 2001; Jacobsen et al., 2003; Maess et al., 2007; Lohvansuu et al., 2013), but this has been less well resolved in animal studies (for supportive evidence in animal models, see, e.g., Astikainen et al., 2011; Nakamura et al., 2011; Parras et al., 2017; Kurkela et al., 2018; Polterovich et al., 2018; for no support or partial support, see, e.g., Fishman and Steinschneider, 2012; Lipponen et al., 2019; Yang et al., 2019). In the many-standards control condition, in addition to the original deviant and standard stimuli, other stimuli with different stimulus features than those in the standard and deviant stimuli are randomly presented but without consecutive repetitions. Each stimulus’s probability is the same as the probability of the deviant stimulus in the oddball paradigm. The many-standards condition is more difficult to design for the somatosensory than for the auditory and visual modality. For instance, with a deviant probability of 10%, this condition would require 10 different stimulation locations for a location-change paradigm in the somatosensory modality, and different skin locations have also different sensitivities. However, to our knowledge, no previous studies have applied this type of experiment in the somatosensory domain in human participants, and only one study in animals is reported (whisker stimulation in rats: Musall et al., 2017).

Here, we introduce a novel modified oddball paradigm that approaches the topic from a different angle. Because it is more difficult in the somatosensory than in the auditory studies to produce several feature levels (such as different frequencies of tones) for application in the many-standards condition, we developed a stimulus condition in which somatosensory responses to equally rare unpredictable and predictable stimuli can be investigated. In this stimulus paradigm, the frequently presented standard stimulus (the frequent stimulus, FRE) is rarely and randomly replaced by a deviant stimulus (the unpredictable rare stimulus, UR), as in the classical oddball paradigm. However, another deviant stimulus (the predictable rare stimulus, PR) immediately follows each UR. Therefore, these two rare somatosensory events are different in their prediction error value, but similar in rareness (probability). The UR should thus show increased responses in comparison to the FRE and PR due to its larger prediction error potential.

In this study, the stimulation is presented as electrical stimulations of fingers, and the three stimulus types differ in location of the stimulation. Consistent with previous studies investigating the location deviance detection and where the fingers or hands have been stimulated in an ignore condition (Shinozaki et al., 1998; Akatsuka et al., 2005, 2007a,2007b; Restuccia et al., 2007; Strömmer et al., 2014, 2017; Hautasaari et al., 2019), we expect that the stimulation will elicit activity in two main time windows at approximately 30–70 and 100–200 ms after the stimulus onset. We also expect both the early and later responses to show a larger amplitude to rare stimuli in comparison to standard stimuli (Mima et al., 1998; Akatsuka et al., 2005, 2007a,2007b; Strömmer et al., 2017; Hautasaari et al., 2019). Since previous studies have not controlled for stimulus rarity (for example, by using the many-standards control condition), we cannot predict whether increased responses in comparison to the FRE will be elicited by the UR alone or by both the UR and the PR. However, larger responses specific to the UR will reflect prediction error, while larger responses to both the UR and the PR would reflect stimulus rarity in comparison to the FRE.

## Materials and Methods

### Participants

Fifteen healthy participants (12 females and 3 males, aged 21–43 years old) were recruited via email lists and notice boards within the University of Jyväskylä and by an announcement in a local newspaper. Inclusion criteria were an age of 18–45 years, right-handedness, and self-reported normal senses (vision corrected with eyeglasses was allowed). Hearing ability for 1,000 and 500 Hz sounds was measured in the laboratory with an audiometer to ensure proper hearing because we also collected another dataset in the auditory sensory modality, not reported here. Exclusion criteria were pregnancy, breastfeeding, current or previous neurological or psychiatric diseases, brain damage, alcohol abuse or use of illegal drugs, and current depressive symptoms. A Finnish-language version of the Beck Depression Inventory II (BDI-II) questionnaire (Beck et al., 1996) was filled in by participants, and a maximum score of 10 in the BDI-II was allowed for included participants. In addition, participants with contraindications for MEG measurement such as a pacemaker, hearing aid, or dental implant were excluded. Before the experiment, a phone interview was conducted to confirm the inclusion and exclusion criteria. Each participant received one movie ticket as compensation for their participation. The experiment complied with the Declaration of Helsinki and was approved by the ethics committee of the University of Jyväskylä. Written informed consent was signed by each participant upon their arrival to the laboratory.

### Stimulus and Task Procedure

Stimuli were electrical pulses (Stimulator: DeMeTec SCG30, DeMeTec GmbH, Langgöns, Germany) of 200 μs in duration, delivered via flexible, non-magnetic metal ring electrodes (Technomed Europe Ltd., Maastricht, Netherlands) to the left forefinger and little finger and stimulating the cathode above the proximal phalanx and the anode above the distal phalanx. All the ring electrodes were moistened with conductive jelly (Technomed Europe Ltd., Maastricht, Netherlands) to reduce impedance. A piece of gauze was tied to the stimulated finger between the two electrodes to prevent conduction between the two electrodes on the same finger. The stimulation intensity was adjusted separately according to the threshold of each finger for each subject. The threshold was determined by the participants’ oral reports when they sensed an electrical pulse. The stimulation started from very low intensity and gradually continued to a higher intensity in increments of 0.1 mA until the participant reported feeling the stimulation. This process was repeated three times and applied to the two stimulated fingers. The intensity applied in the experiment was 1.5 times the subjective sensory threshold intensity.

**FIGURE 1:**
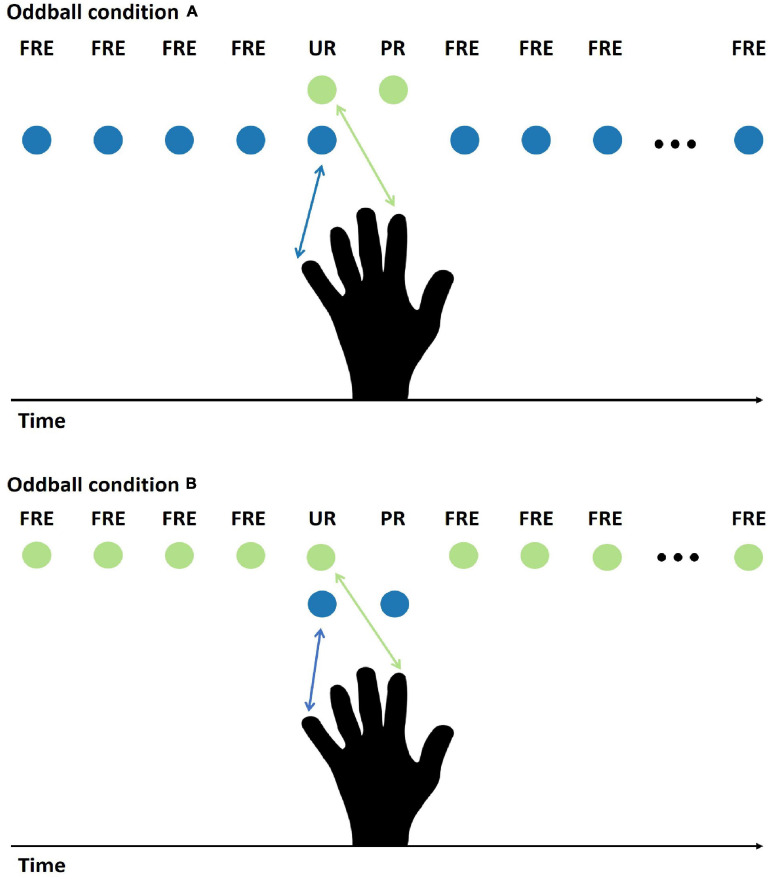
Illustration of the stimulus presentation for conditions A and B. Under condition A, stimulation to the little finger (blue ball symbol) served as the FRE, stimulation to the forefinger (green ball symbol) as the PR, and simultaneous pulses to the forefinger and the little finger as the UR. Under condition B, the opposite assignment between the FRE and the PR was applied. In the analysis, conditions A and B were averaged; therefore, the physical features of the FRE and the PR are controlled. FRE, frequent stimulus; UR, unpredictable rare stimulus; PR, predictable rare stimulus.

The stimulus procedure was a modified oddball paradigm. A frequently presented stimulus was occasionally replaced by two different rare stimuli: the first one, which was unpredictable, was always followed by another one that was predictable. The experiment had two main stimulus conditions (condition A and condition B, Figure 1), which had counterbalanced stimulus features for the FRE and the PR. In condition A, the FRE was stimulation to the little finger, and the PR was stimulation to the forefinger. In condition B, the stimulus assignment was reversed for the FRE and PR. The unpredictable rare stimulus (UR) was a double stimulation (forefinger and little finger, simultaneously). The double stimulation was selected because we did not want to stimulate an additional finger, which would have been necessarily adjacent to either little finger or forefinger. This is because it is not known whether stimulation of adjacent fingers elicits differential responses, but we know from our previous studies that stimulation of the little finger and forefinger can elicit a differential response between the deviant and the standard stimuli (Strömmer et al., 2014, 2017). In addition, not stimulating additional fingers can also avoid the potential boundary effect. This is because previous studies have shown a significantly larger sMMR contrast between the middle finger and the thumb than between the middle finger and the little finger (Shen et al., 2018). Therefore, applying stimulation to additional fingers could also introduce other possible stimulus features variance.

In order to counterbalance the physical features of the stimuli for sMMR assessment, an additional experiment with condition C was conducted for four participants after the presentation of conditions A and B. In condition C, the FRE was a stimulation of the forefinger and little finger, simultaneously, whereas the UR and PR were stimulations to the forefinger and little finger, respectively (see Supplementary Material 1 for the experimental setting and results). Therefore, when averaging the responses of conditions B and C, the stimulus features were counterbalanced for the FRE and the UR.

Each condition consisted of 1,000 trials presented in two runs for each participant. The probability of an FRE was 80%, and the probability of a UR or PR was 10%. The presentation order of the runs was counterbalanced between the participants, and a short break was provided after each run. The interstimulus interval (ISI, offset-to-onset) was 500 ms under all conditions. The stimulus presentation was controlled by Presentation^®^ software (Neurobehavioral Systems, Inc., Berkeley, CA, United States). Participants were instructed to ignore the somatosensory stimuli and focus on a silent movie. The movie was projected onto the center of the screen at a distance of about 1 m from the participant (video projector: Barco FL35 projector; native resolution 1,920 × 1,080 pixels).

### Data Acquisition

The somatosensory evoked related magnetic fields were recorded with a 306-channel whole-head system (Elekta Neuromag TRIUX^TM^ system, Elekta AB, Stockholm, Sweden) in a magnetically shielded, dimly lit room at the MEG Laboratory, University of Jyväskylä.

During the MEG recording, the participant was seated on the chair with their head inside the helmet-shaped device at a 68° upright position. The head position with respect to the sensors in the helmet was determined at the beginning of the task according to the magnetic fields produced by currents fed into five indicator coils at predetermined locations on the scalp. Two HPI coils were placed on both sides behind each ear; another three were placed on the forehead. The locations of these coils in relation to the anatomical location of preauricular points and nasion were determined with an Isotrak 3D digitizer (Polhemus^TM^, United States) before the experiment started. More than 100 additional points were digitized over the scalp to provide an accurate representation of the individual head shape and for co-registration with a magnetic resonance imaging (MRI) template. The continuous MEG signal was recorded with an online bandpass filter of 0.1–330 Hz and a sampling frequency of 1,000 Hz. The electrooculogram (EOG) and electrocardiogram (ECG) signals were recorded by detecting eye movements and heartbeat artifacts, respectively. The vertical EOG was recorded by two electrodes attached above and below the right eye; the horizontal EOG was recorded by two electrodes placed on the outer canthi of both eyes. One ECG electrode was placed below the collar bone on the right side, and the other was placed in the middle of the two collar bones. A ground wristband was wrapped around the participant’s left-hand carpal bone.

### Data Analysis

The Maxfilter 3.0 (Elekta AB) was first applied to reduce the artifacts and transform the mean head positions across different recording sessions. Bad channels were marked manually. The spatiotemporal signal space separation (tSSS) method (Taulu et al., 2004), with a buffer of 30 s and a subspace correlation limit of 0.98, was used to remove external interference from the data. The head position was estimated for head movement compensation with the default setting (HPI amp window: 200 ms; HPI amp step: 10 ms).

The MEG data were then preprocessed and analyzed using the Brainstorm software (Tadel et al., 2011). First, a notch filter of 50 Hz (3 dB notch bandwidth: 2 Hz) and a low-bandpass filter of 60 Hz were applied, as described previously (Hautasaari et al., 2019). Cardiac and eye blink artifacts were attenuated with signal space projection (SSP) in Brainstorm by visually inspecting and removing the corresponding SSP components separately for gradiometers and magnetometers. Additionally, data with EOG amplitudes exceeding 200 μV were marked as bad. The data were then made into epochs according to the stimulus events from a 100 ms pre-stimulus baseline to 500 ms from the stimuli onset. A DC offset baseline correction of -100 to 0 ms was calculated and removed for each epoch. Epochs that included a segment in which the EOG amplitudes exceeded 200 μV were rejected.

The responses were then averaged for each stimulus type over condition A and condition B (weighted average with the number of trials in each condition). Only FRE responses immediately preceding the UR were applied in the analysis because this allowed an equal number of trials for each stimulus type. Conditions A and B were then combined to counterbalance the physical properties of the FRE and the PR. More specifically, a weighted average based on the number of trials was calculated for the rare (both UR and PR) and the FRE responses across conditions A and B for each participant.

For sensor-level comparisons, planar gradiometer channel pairs were combined using root mean squares (RMSs) at each sensor location. For source-level analysis, because individual MRI data were not available, the FSAverage_2016 anatomy template from Brainstorm was used for the MRI co-registration and further source analysis. To make the template better match each participant’s head shape, we warped the anatomy templates to match the shape defined by the digitized points. The noise covariance matrix was estimated from an empty room recording made on the same day or on neighboring days. For the MEG forward model, the sensor-weighted overlapping sphere model (one per sensor, in a total of 306 local spheres) (Huang et al., 1999) was used for the representation of the cortical surface with 45,000 dipoles (3 orientations × 15,000 vertices). The inverse solution was performed using the unconstrained depth-weighted minimum-norm estimates (wMNE) implemented in Brainstorm. The unconstrained wMNE were used to avoid the possible noisy and discontinuous current maps since we used the anatomy template instead of individual MRI data for the source estimate. The source localization results were then normalized with a *Z*-score based on the baseline from -100 to 0 ms relative to the stimulus onset. The norm of the three orientations for the unconstrained source was used in the subsequent analysis.

### Statistical Analysis

**FIGURE 2:**
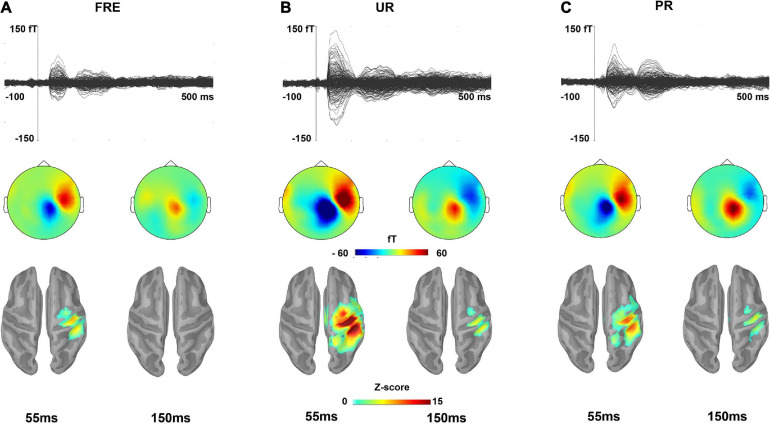
The grand-averaged responses for **(A)** FRE, **(B)** UR, and **(C)** PR. The upper panel shows a butterfly view of the grand-averaged response for each stimulus type from all 306 sensors. For visualization purposes, gradiometer values are multiplied by 0.04 due to the differing units for magnetometers (T) and gradiometers (T/m). The middle and lower panels show the topographies of the sensor-level activity based on magnetometers and source activation, respectively, at a time point of 55 and 150 ms for each stimulus type. In the lower panel, only the sources that have a value > 40% of the color bar maximum are displayed. FRE, frequent stimulus; UR, unpredictable rare stimulus; PR, predictable rare stimulus.

Sensor-level analyses were carried out in Brainstorm by calling the spatiotemporal cluster-based permutation test functions from the Fieldtrip toolbox (Maris and Oostenveld, 2007). Since the results were similar to the source-level results, the detailed statistical analysis and main results of the sensor-level data are reported in Supplementary Material 2. Previous MEG studies in the ignore condition have suggested that sMMR is mainly elicited in the primary somatosensory cortex (SI) and the secondary somatosensory cortex (SII) (e.g., Akatsuka et al., 2007a,b; Naeije et al., 2016, 2018; Hautasaari et al., 2019). Thus, based on these prior findings and verified in our grand-averaged source maps of the UR and PR (Figure 2), we defined two regions of interest (ROIs), namely, SI (G_postcentral: postcentral gyrus) and SII (Lat_Fis-post: posterior ramus of the lateral fissure), based on the Destrieux atlas (Destrieux et al., 2010). Moreover, only the regions on the right hemisphere, which mean the contralateral SI (cSI) and the contralateral SII (cSII), were used since little or no activation occurs in the corresponding brain regions on the left hemisphere (Figure 2) (for previous studies in which only the contralateral side was activated, see, e.g., Strömmer et al., 2014, 2017; Naeije et al., 2016, 2018). The norms of the three orientations for an unconstrained source within the same time windows (30–100 and 130–230 ms after stimulus onset) used in the sensor-level analysis were exported from Brainstorm into the SPSS program for further analysis. For each identified ROI and time window, a separate one-way repeated-measures analysis of variance (ANOVA), with stimulus type (FRE, UR, and PR) as the within-subjects factor, was conducted. The Greenhouse–Geisser correction [*p*-value after Greenhouse–Geisser correction (*p*_*corr*_)] was applied when the assumption of sphericity was not met. For significant ANOVA results, *post hoc* analyses were conducted by using a two-tailed paired *t*-test with different stimulus type pairs. Partial eta squared (η^2^_*p*_) measures were used for effect size estimates in ANOVA. Bonferroni correction was used for both ANOVA and *post hoc* analysis to control for the multiple comparison problem [*p*-value after Bonferroni correction (*p*_*corr*_)]. Cohen’s (1988) *d* was computed with pooled standard deviations for the effect size estimate in the *t*-test.

## Results

### Descriptive Results

**FIGURE 3:**
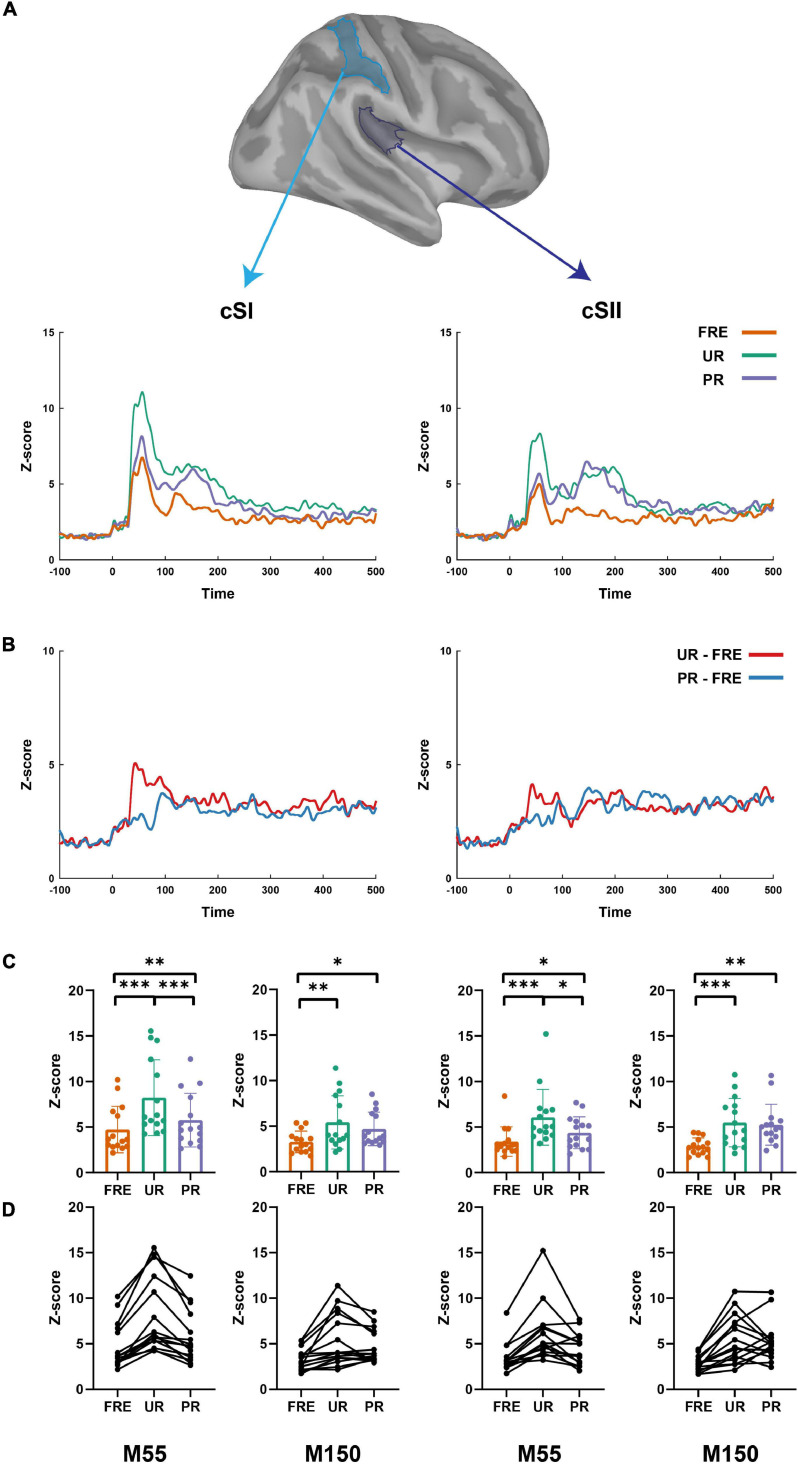
The summary of the results from the source-level analysis. **(A)** The cortical time series for all three conditions (UR, PR, and FRE) in the cSI (left panel) and cSII (right panel). **(B)** The cortical time series for the differential responses (UR–FRE and PR–FRE) in the cSI (left panel) and cSII (right panel). **(C)** The bar graph of the source strength comparison of the FRE, UR, and PR in the cSI (left panel) and cSI (right panel). Error bars represent the standard error of the mean, and the dots represent the values of the individual participants. **(D)** Line graphs of the individual participant’s source strengths to the three stimulus types in the cSI (left panel) and cSII (right panel). FRE, frequent stimulus; UR, unpredictable rare stimulus; PR, predictable rare stimulus; ^∗^*p* < 0.05, ^∗∗^*p* < 0.01, and ^∗∗∗^*p* < 0.001.

Figure 2 illustrates the grand-averaged sensor-level responses and the source estimates for the FRE, UR, and PR. Figures 3A,B illustrate the source activity waveform on both ROIs for each stimulus type (UR, PR, and FRE) and differential responses (UR–FRE and PR–FRE), respectively. As shown in Figures 2, 3A, the response waveforms are characterized by two main components: one at approximately 30–100 ms latency (M55) and the other at approximately 130–230 ms latency (M150). The corresponding topography and source activation for each component are also presented in Figure 2. The sensor-level results are reported in Supplementary Material 2.

### Source Activations

#### M55

**FIGURE 4:**
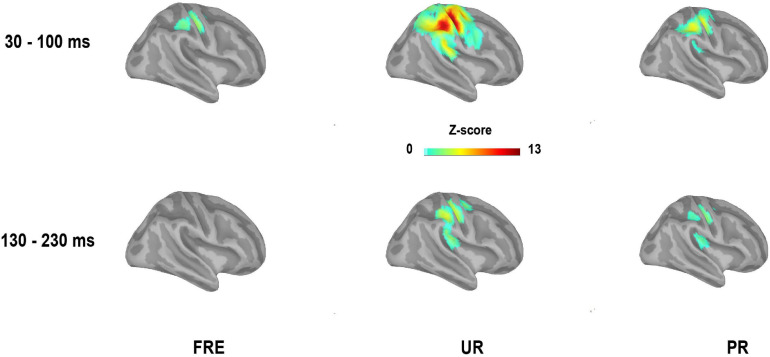
Grand-averaged source activation for the time window of 30–100 ms (M55) and 130–230 ms (M150) after stimulus onset for each stimulus type from the right-side view (mean values of the time windows presented). For visualization purposes, only the sources with a value > 40% of the color bar maximum are displayed here. FRE, frequent stimulus, UR, unpredictable rare stimulus; PR, predictable rare stimulus.

For the results of the mean source activation value in 30–100 ms latency, one-way repeated-measures ANOVA showed main effects of stimulus type in both the cSI and cSII: in the cSI, *F*(2,28) = 32.049, *p*_*corr*_ < 0.001, η^2^_*p*_ = 0.696); in the cSII, *F*(2,28) = 18.126, *p*_*corr*_ < 0.001, η^2^_*p*_ = 0.564. *Post hoc* paired *t*-tests with Bonferroni-corrected *p-*values are reported in Table 1 and Figure 3C. *Post hoc* tests revealed that both the PR and UR showed increased activation compared to the FRE in both the cSI and the cSII areas. In addition, both ROIs showed an increased source strength for the UR compared to the PR. The line graph of individuals’ source strength to the three stimulus types are illustrated in Figure 3D. The grand-averaged source activations for different stimuli from the right-side view are illustrated in Figure 4.

**TABLE 1:** *Post hoc* paired-samples *t*-tests investigating the main effect of the stimulus type found in the repeated-measures ANOVA for M55.

**Conditions**	**cSI**			**cSII**		
	***t***	***p*_*corr*_**	***d***	***t***	***p*_*corr*_**	***d***
PR vs. FRE	4.121	0.003	0.376	3.199	0.019	0.576
UR vs. FRE	6.612	<0.001	1.014	6.175	<0.001	1.086
UR vs. PR	4.816	<0.001	0.685	2.977	0.030	0.677

*PR, predictable rare stimulus; FRE, frequent stimulus; UR, unpredictable rare stimulus; cSI, contralateral primary somatosensory cortex; cSII, contralateral secondary somatosensory cortex; *p*_*corr*_, *p*-value after Bonferroni correction; *d*, Cohen’s *d*. The degrees of freedom for all comparisons are 14.*

#### M150

For M150, significant main effects for the stimulus type were found in both ROIs; cSI: *F*(2,28) = 11.355, *p* < 0.001, η^2^_*p*_ = 0.448); cSII: *F*(2,28) = 14.798, *p* < 0.001, η^2^_*p*_ = 0.514. *Post hoc t*-tests are reported in Table 2 and Figure 3C. The results showed that in both ROIs, both the PR and the UR induced larger activity compared to the FRE. However, no difference was found between the UR and the PR in either the cSI or the cSII areas. The line graph of individuals’ source strength to the three stimulus types are illustrated in Figure 3D. The grand-averaged source activations for the different stimuli from the right-side view are illustrated in Figure 4.

**TABLE 2:** *Post hoc* paired-samples *t*-tests investigating the main effect of stimulus type found in the repeated-measures of ANOVA for M150.

**Conditions**	**cSI**			**cSII**		
	***t***	***p*_*corr*_**	***d***	***t***	***p*_*corr*_**	***d***
PR vs. FRE	3.528	0.010	0.921	4.357	0.002	1.381
UR vs. FRE	3.768	0.006	0.962	5.161	<0.001	1.315
UR vs. PR	1.905	0.232	0.294	0.434	1.000	0.095

*PR, predictable rare stimulus; FRE, frequent stimulus; UR, unpredictable rare stimulus; cSI, contralateral primary somatosensory cortex; cSII, contralateral secondary somatosensory cortex; *p*_*corr*_, *p*-value after Bonferroni correction; *d*, Cohen’s *d*. The degrees of freedom for all comparisons are 14.*

## Discussion

In the present study, we introduced a new oddball stimulus protocol for investigating brain responses to unpredictable and predictable rare somatosensory events. Use of this stimulus protocol allowed us to control for the rarity (probability) of the unpredictable and predictable stimuli. We found two main components, M55 and M150, for each stimulus type: the frequent stimulus (FRE), unpredictable rare stimulus (UR), and predictable rare stimulus (PR). The sources of both components were located on the contralateral somatosensory cortices. The sensor-level (see Supplementary Material 2 for a detailed report) and the source-level results showed a similar pattern: both components elicited a larger activity for the UR and PR than for the FRE. A larger response was observed for the UR than for the PR only for M55, whereas no difference was found in response amplitudes between the UR and the PR for M150. This pattern of results suggests that M55, but not M150, possibly signals the prediction error.

The latencies of the components, one at 30–100 ms latency (M55) and the other at 130–230 ms latency (M150), were well in line with the previous MEG studies that have found an early component approximately at 30–70 ms latency and a later component at approximately 100–200 ms after stimulus onset (Mima et al., 1998; Akatsuka et al., 2007a,b; Hautasaari et al., 2019). Some EEG studies that applied the somatosensory oddball paradigm have also found two components with similar latencies as M55 and M150 here (Shinozaki et al., 1998; Akatsuka et al., 2005; Restuccia et al., 2007; Strömmer et al., 2014, 2017). Consistent with previous MEG oddball studies that applied source localization (Mima et al., 1998; Akatsuka et al., 2007a,b; Naeije et al., 2016, 2018; Hautasaari et al., 2019), both components were elicited on the sensory cortices (SI and/or SII).

Our results resemble those of the previous somatosensory studies that applied a traditional oddball paradigm to elicit the sMMR; however, our data raise questions regarding the interpretation of the previous studies that the responses to rare unpredictable stimuli (here UR) at 100–200 ms latency reflect a prediction error (e.g., Mima et al., 1998; Shinozaki et al., 1998; Akatsuka et al., 2005, 2007a; Strömmer et al., 2014, 2017; Hautasaari et al., 2019). Namely, when we used equally rare stimuli with different types of predictability (UR and PR), the responses to these two stimuli did not show any amplitude difference for M150, but they did for M55. Although several studies have found larger responses to deviant than to standard stimuli at early latency (within the 100 ms post-stimulus latency, Mima et al., 1998; Shinozaki et al., 1998; Akatsuka et al., 2005,2007a,2007b; Strömmer et al., 2014, 2017; Yamashiro et al., 2014; Hautasaari et al., 2019), these studies have usually considered only the later response (between 100 and 200 ms post stimulus), but not the earlier one (before 100 ms) as being analogous to sMMR (e.g., Mima et al., 1998; Shinozaki et al., 1998; Akatsuka et al., 2005, 2007a; Strömmer et al., 2014, 2017; Hautasaari et al., 2019). However, they did not provide any empirical evidence for the assumption of the specificity of the later response to a prediction error, nor did they rule out the effect of stimulus rareness (for example, by applying the many-standards control condition). Therefore, the previous findings of differential responses to deviant stimuli at 100–200 ms post-stimulus latency may possibly have reflected merely the rareness of the deviant stimulus. Conversely, the differential responses at the earlier latency (before 100 ms) reported in the previous studies (Mima et al., 1998; Shinozaki et al., 1998; Akatsuka et al., 2005, 2007a,2007b; Strömmer et al., 2014, 2017; Yamashiro et al., 2014; Hautasaari et al., 2019) could reflect a prediction error. Notably, the results from a previous MEG study indicated that two components, one at 30–70 ms and the other at 150–250 ms latency, showed increased amplitudes to deviant stimuli presented at 10%, but not at 30 or 50% probability (Akatsuka et al., 2007b). The results of this previous study, together with those of our study in which the predictability of the rare stimulus was manipulated, suggest that the earlier MEG component (here M55) could be specific to the prediction error and that the later responses (here M150) might reflect merely the stimulus rareness. Furthermore, studies that used a global/local paradigm to verify the hierarchical processing network of the sMMR at different levels found that a response peaking at 70–100 ms over the posterior bank of the postcentral sulcus reflected the prediction error (Naeije et al., 2016, 2018). In rabbits, similar and even earlier latencies (i.e., 20–40 and 80–100 ms) for somatosensory deviance detection have been found in recordings of local-field potentials from the somatosensory cortex (deviant-alone control condition, Astikainen et al., 2001).

Not only some of the previous studies in the somatosensory modality but also those in the auditory modality have reported deviance detection at early latencies. For example, the auditory middle latency responses (MLRs), elicited within 50 ms latency after the stimulus onset, have been studied in the context of predictive coding (e.g., Althen et al., 2011; Grimm et al., 2011; Recasens et al., 2014). These responses have their source generator possibly in the sensory cortex (Recasens et al., 2014), and a recently suggested view (Grimm et al., 2016) is that the MLRs could be correlates of stimulus-specific adaptation (SSA, Ulanovsky et al., 2003), which also occurs in a similar latency range. SSA (i.e., adaptation to repeated sounds that do not generalize to other sounds) is widely studied in animals with single-cell recordings. Although the name of the phenomenon refers to adaptation, release from SSA can also support genuine deviance detection (e.g., Parras et al., 2017; for a review, see Carbajal and Malmierca, 2018). Interestingly, a rat study that contrasted the auditory cortical responses to patterns of periodic (predictable) and random (unpredictable) changes in sounds found larger intracellular and extracellular responses to random than to periodic changes (Yaron et al., 2012). Future studies using both single-cell and neural network-level recordings are needed to understand whether the early latency brain responses (e.g., MLRs and the M55 reported here) in the auditory and somatosensory modalities have functional similarities and whether they share neural mechanisms for rareness and/or deviance detection.

Here, the activity for both the UR and the PR was most pronounced on the sensory cortices (i.e., the SI and SII). Although some discrepancies exist regarding whether the activity has been found from the SI, the SII, or both, previous studies applying the somatosensory oddball condition have mainly located deviance detection-related responses in the SI and/or SII. Akatsuka et al. (2007a, b), who first applied the source localization method for the sMMR, suggested that the early component (30–70 ms) originates mainly from the SI. The later component (150–250 ms) was located mainly in the SI, but the data from some individuals showed the generators in the SII (Akatsuka et al., 2007a,b). Later, areas 1 and 3b of the SI, as well as the posterior parietal cortex (PPC), were linked to the deviance detection at approximately 50–120 ms post-stimulus latency. Deviance detection-related activity was also found on the bilateral SII cortex in a few participants (Yamashiro et al., 2014). Both the electrical and tactile stimuli also elicited SI activity for the early component (40–58 ms), and SII activity for the later component (110–185 ms) (Hautasaari et al., 2019). Some studies have also found simultaneous SI and SII responses as early as 20–30 ms (Karhu and Tesche, 1999) instead of a strict hierarchical or serial manner, suggesting that the SI and SII could process somatosensory stimuli in a parallel manner. Taken together with our results, the available evidence indicates a likelihood that the SI and SII could both contribute to the deviance detection and could also possibly be linked to the prediction error.

Even if our study strongly suggests that the increased response amplitude for M150 does not reflect a prediction error, the current study is limited in its interpretation regarding M55. The M55 was larger in amplitude for the UR than for the FRE and PR; however, whether the increased response amplitude reflects the prediction error or a larger stimulus energy for the UR in comparison to the PR and FRE is unclear. This is because the low-level stimulus features were not counterbalanced for all the stimulus types, but only between the FRE and PR. The stimulus energy for the UR (stimulation of two fingers at the same time) was larger than for the PR and FRE (stimulation of one finger) when the data combined from conditions A and B were analyzed. Therefore, we conducted an additional measurement (condition C) for a small subsample of participants (*n* = 4). In this measurement, the physical characteristics of the UR and FRE were reversed for condition B (Supplementary Material 1). Thus, when the data were combined from conditions B and C, the responses to the UR and FRE were counterbalanced for their low-level features. Visual observation of the data suggests that three of the four participants showed numerically larger activity for the UR than for the FRE in the M55 time range, and two of the four participants showed the same for M150. This suggests that the difference in low-level physical features was probably not the only reason for the larger responses to the UR than to the FRE in the larger sample, and this tentatively associates M55 with the prediction error.

Our paradigm may also be applied to the other sensory modalities. In the auditory modality, the many-standards control condition has recently been the most commonly used protocol to control for the effect of stimulus probability (e.g., Jacobsen and Schröger, 2001, 2003). However, the results may be affected by the cross-frequency adaptation (Taaseh et al., 2011) between the oddball and control condition sounds. The cross-frequency adaptation is usually observed as a reduced response amplitude to consecutive sounds of nearby frequencies. Because more sounds are present, and usually with smaller frequency differences in the control than in the oddball condition, the responses can be larger to the oddball deviant sounds than to the control sounds merely for this reason (see discussion in Yang et al., 2019, where the oddball and many-standards conditions have the same frequency separation in rats). The novel paradigm introduced in the present study can avoid this problem, because it does not require many different stimuli, and the stimuli can also be clearly distinct in frequency (or other changing feature). However, all three stimulus conditions (here conditions A and B in Figure 1 and condition C in Supplementary Material 1) are required to fully counterbalance the physical features of the three stimuli.

In summary, our results suggest that the processing of a stimulus site change in the electrical stimuli on the fingers induces two main components: M55 and M150. M55 was larger for the UR than for the FRE and PR over both the SI and SII. Surprisingly, although the UR had a larger prediction error potential and an even larger stimulus energy than the PR, it did not show an increased M150 amplitude when compared to the PR. Our data therefore tentatively link M55, but not M150, to signaling of the prediction error. The results also highlight the need for controlling the stimulus rareness or for disentangling stimulus rareness and predictability in future studies.

## Data Availability Statement

The raw data supporting the conclusions of this article will be made available by the authors, without undue reservation.

## Ethics Statement

The studies involving human participants were reviewed and approved by the Ethical Committee of the University of Jyväskylä. The patients/participants provided their written informed consent to participate in this study.

## Author Contributions

QX and PA conceived the experiments. QX, ER, and XL performed the data acquisition. QX analyzed the data. JH contributed to data analysis. QX, CY, JH, and PA interpreted the data. QX, CY, and PA drafted the manuscript. JH, ER, and XL provided critical revisions. All authors revised and approved the manuscript.

## Conflict of Interest

The authors declare that the research was conducted in the absence of any commercial or financial relationships that could be construed as a potential conflict of interest.
