# Supplementary material for: Magnetoencephalography Responses to Unpredictable and Predictable Rare Somatosensory Stimuli in Healthy Adult Humans
Source: Front Hum Neurosci. 2021 Apr 14;15:641273. doi: 10.3389/fnhum.2021.641273 (PMC8079819; doi:10.3389/fnhum.2021.641273)
Supplement: Supplementary file 1 [file Data_Sheet_1.pdf]

Supplementary Material 1

Magnetoencephalography responses to unpredictable and predictable rare somatosensory stimuli in healthy adult humans

1 Introduction: additional data controlling for physical features of the stimuli in the analysis of the sMMR

In our study reported in the main text, the focus was on comparison of the responses to repetitive (FRE) and predictable rare (PR) stimuli with the aim of demonstrating the effect of rareness without prediction error. Therefore, the stimulus conditions A and B were counterbalanced for these stimulus types. However, the stimuli that elicited a mismatch response (i.e., standard and unpredictable rare stimuli) were not counterbalanced under these conditions.

To estimate to what extent the stimulus characteristics affected the responses to the UR, especially because the UR was larger in energy than the FRE, we conducted an additional recording, condition C, in four of the original fifteen participants who were available for the measurement. Due to an insufficient number of participants for group-level statistical analysis, we report here the descriptive figures of the averaged responses with a 95% confidence interval (CI) derived from single trials for each participant.

2 Method

The participants were four females, aged 22−31 years old. In condition C, the FRE and UR were reversed to that of condition B (Supplementary Material 1, Figure 1). We combined the responses from conditions B and C in the analysis, allowing counterbalancing of the physical properties of the FRE and UR in the responses.

All the preprocessing methods for condition C were identical to those described in the main text for conditions A and B. Only sensor level analyses were conducted, and they are presented below. Like the results reported in the main text, the following results for the UR and FRE were extracted from the root mean square of the paired gradiometer in two orthogonal directions from the same sensor location. The somatosensory mismatch response (sMMR) is defined here as a difference between the responses to the UR and FRE (UR-FRE). All the waveforms present in the following figures were averaged from the most pronounced channel cluster, including eight sensors; that is, it is the same as in the Supplementary Material 2 reported for the whole sample (Supplementary Material 2, Figure 1A).

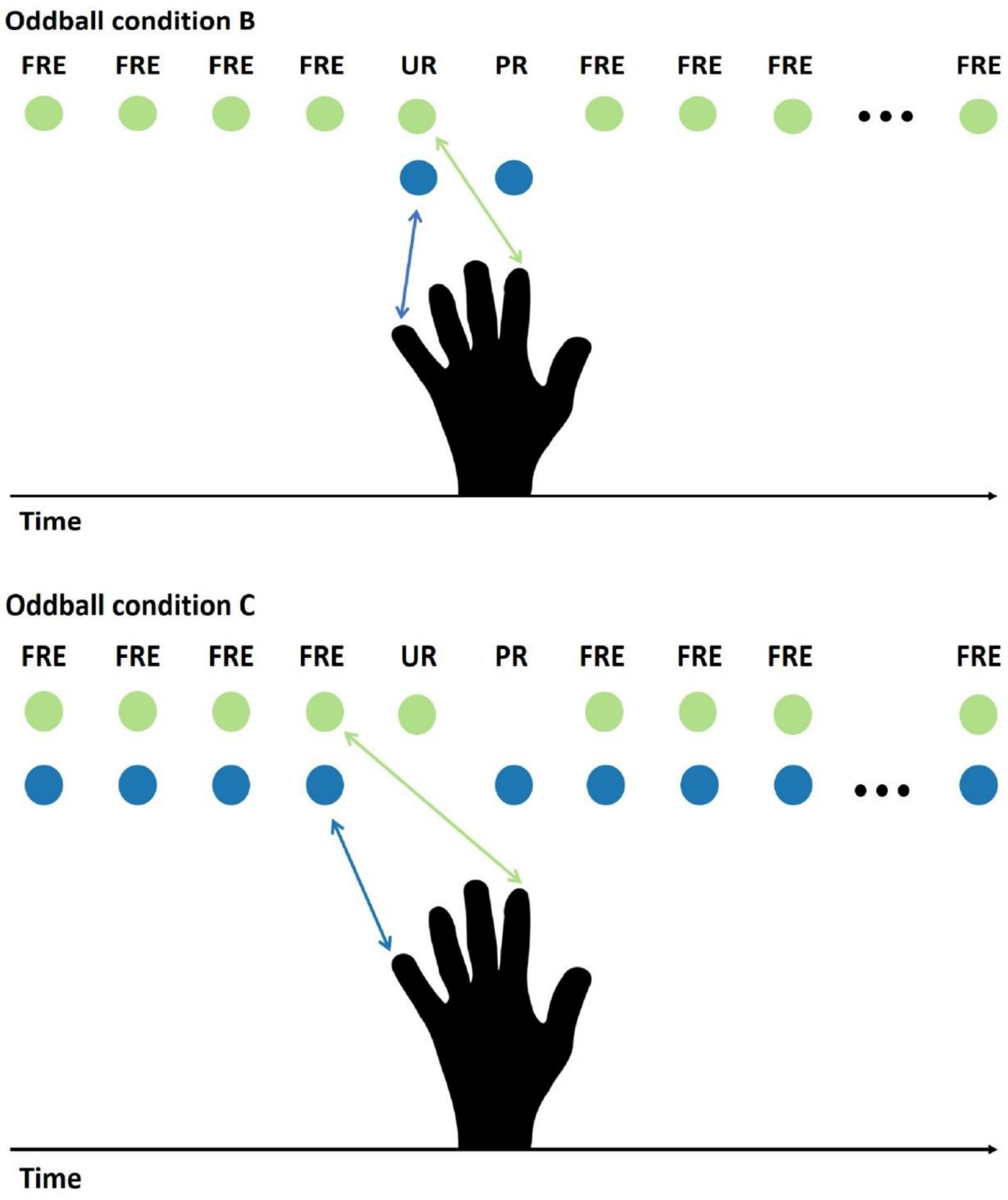

Figure 1. Illustration of the stimulus presentation for conditions B and C. In condition B, the stimulations to the forefinger (green ball symbol) and little finger (blue ball symbol) served as the frequent (FRE) and predictable rare (PR) stimuli, respectively, and the simultaneous stimulation to the forefinger and little finger served as the unpredictable rare (UR) stimulus. By contrast, in condition C, the FRE stimulus was manifested as the simultaneous stimulation of the forefinger and the little finger. The UR and PR were applied to the forefinger and little finger, respectively.

3 Results

Supplementary Material 1, Figure 2 shows the grand-averaged results for the four participants. Visual inspection of the grand-averaged waveforms indicates that both the UR and FRE show two main components corresponding to the M55 (30–100 ms) and M150 (130–230 ms) reported in the main text. The UR seems to induce a larger amplitude than the FRE in both time windows.

Supplementary Material 1, Figure 3 shows the individual participants' responses (condition B and C, averaged). Visual inspection of the waveforms suggests that all four participants show the two investigated components (corresponding to M55 and M150) for the FRE and UR stimuli. Furthermore, for three participants (participants 1, 3, and 4), the responses are numerically larger in amplitude for the UR than for the FRE in the earlier time window, while two participants (participants 1 and 2) show a larger response to the UR than to the FRE in the later time window.

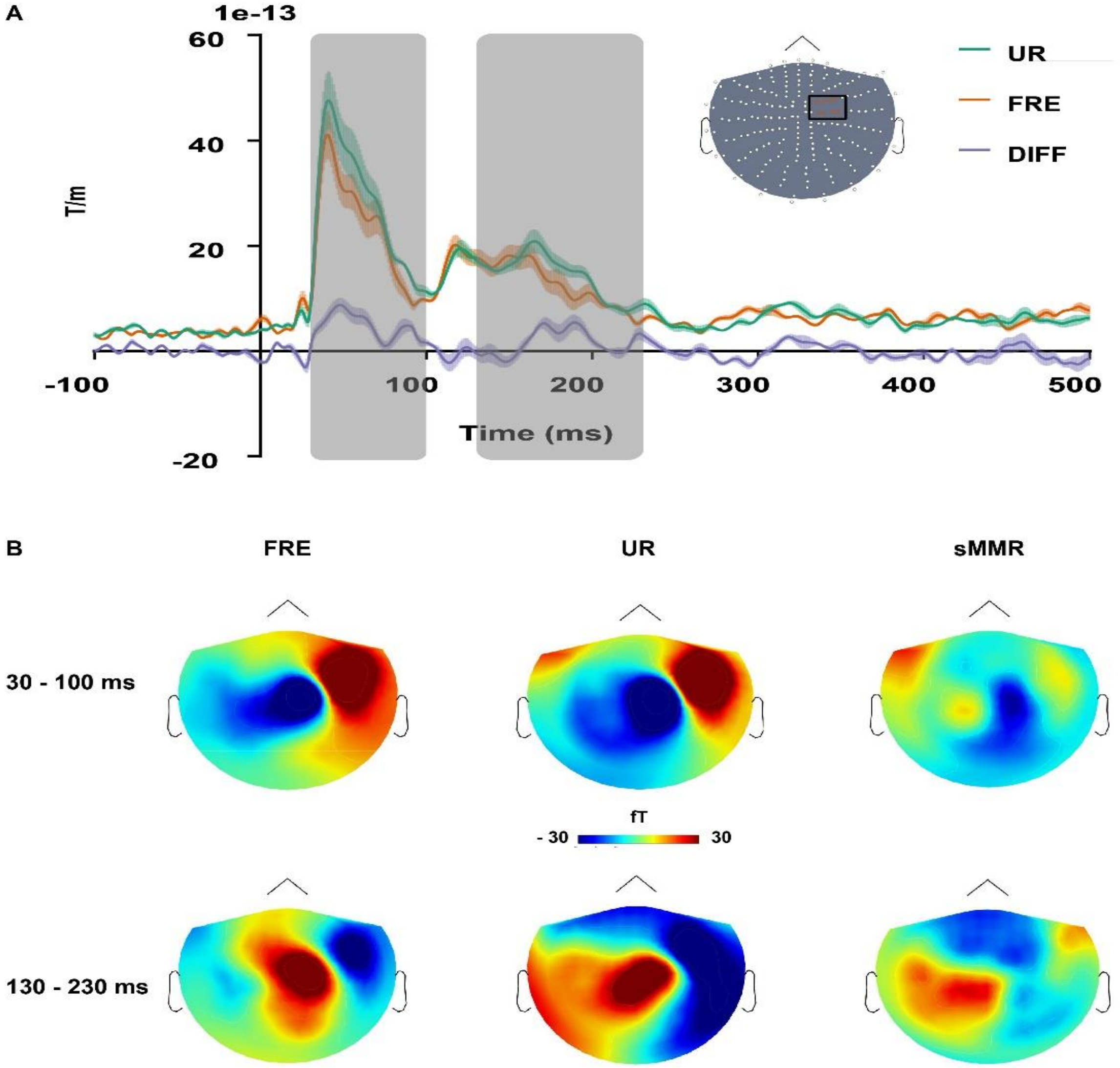

Figure 2. Sensor level grand-averaged results of four participants (conditions B and C averaged, thus physical features for the FRE and UR counterbalanced). (A) Grand-averaged waveforms (n = 4) with 95% CI averaged from the eight most pronounced sensors (marked with red dots in the black frame in the sensor map). Orange line: frequent stimulus (FRE); Green line: unpredictable rare stimulus (UR); Purple line: sMMR (somatosensory mismatch response, obtained by subtracting the FRE from the UR). The gray shaded areas indicate the time window for M55 (30–100 ms) and M150 (130–230 ms) analyzed in the main text. (B) Topographical maps of the FRE, UR, and sMMR for M55 and M150 extracted as mean amplitude values from the time window of 30–100 ms and 130–230 ms after stimulus onset, respectively. *Upper panel*: topography of M55 (30–100 ms); Lower panel: topography of M150 (130–230 ms).

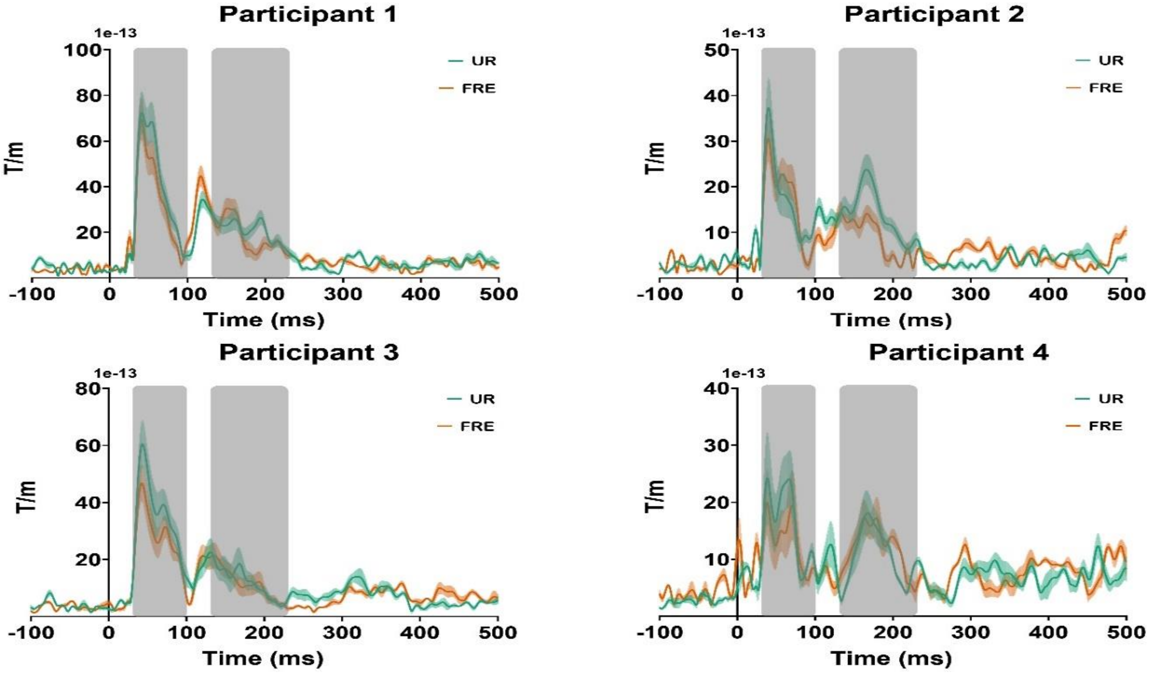

Figure 3. Waveforms (mean and 95% CI) for each of the four participants (the responses are an average of the eight most pronounced sensors). Orange line: frequent stimulus (FRE); Green line: unpredictable rare stimulus (UR). The gray shaded areas indicate a time window of M55 (30–100 ms) and M150 (130–230 ms) applied in the analysis reported in the main text.

As these data stem from only a small sample (n = 4) without statistics, we cannot provide convincing evidence for elicitation of the sMMR. However, visual observation suggests that the responses to the UR are numerically larger than the responses to the FRE for at least some participants. This finding was observed in three of the four participants for M55 and two of the four for M150.
