# Supplementary material for: Magnetoencephalography Responses to Unpredictable and Predictable Rare Somatosensory Stimuli in Healthy Adult Humans
Source: Front Hum Neurosci. 2021 Apr 14;15:641273. doi: 10.3389/fnhum.2021.641273 (PMC8079819; doi:10.3389/fnhum.2021.641273)
Supplement: Supplementary file 2 [file Data_Sheet_2.PDF]

Supplementary Material 2

Magnetoencephalography responses to unpredictable and predictable rare somatosensory stimuli in healthy adult humans

1 Data analysis and statistical analysis at the sensor level

For sensor level comparison, planar gradiometer channel pairs were combined using root mean squares (RMS) at each sensor location. Sensor level analyses were carried out in Brainstorm by calling the spatio-temporal cluster-based permutation test functions from the Fieldtrip toolbox (Maris and Oostenveld, 2007). Time windows for the analysis were restricted based on visual inspection of the maximum of the global field power (GFP) from the grand-averaged response (Supplementary Material 2, Figure 1A) and results of the previous somatosensory studies (e.g., Mima et al., 1998; Akatsuka et al., 2005; Strömmer et al., 2017; Hautasaari et al., 2019). Accordingly, two time windows were selected for further investigation: one at 30–100 ms (labeled as M55) latency and the other at 130–230 ms (labeled as M150) latency after stimulus onset. Over the corresponding time ranges for each component, the contrast between the PR and FRE, the UR and FRE, and the UR and PR were conducted separately in each time window. The channel cluster alpha was set as 0.05; the number of permutations was 1000, with no minimum cluster size determined. This cluster-based permutation test was based on the permutation distribution of the maximum cluster-level sum, which is beneficial in controlling for multiple comparisons.

2 Sensor level results

The results are depicted in Supplementary Material 2, Figure 1.

2.1 M55

Each stimulus pair comparison demonstrated a significant cluster for M55 (30–100 ms). In the PR vs. FRE comparison (PR > FRE, $p = 0.010$, cluster statistic: 936, cluster size: 324, largest cluster found at time point 96 ms after stimulus onset), the difference was most pronounced at sensors over the right parietal and temporal areas at 70–100 ms latency. In the comparison of the UR vs. FRE (UR > FRE, $p = 0.002$, cluster statistic: 7592, cluster size: 2204, the largest cluster found at time point 42 ms after stimulus onset), a significant cluster was found at sensors over the right frontoparietal and temporal areas including the whole time window (30–100 ms). A significant cluster was also found between the responses to the UR and PR (UR > PR, $p = 0.002$, cluster statistic: 5908, cluster size: 1731, largest cluster found at time point 38 ms after stimulus onset), with the difference being most pronounced in the right frontoparietal and temporal areas.

2.2 M150

In the time window of 130–230 ms, corresponding to M150, the cluster-based permutation test revealed spatio-temporal clusters for the PR vs. FRE and the UR vs. FRE. For the PR vs. FRE comparison (PR > FRE, $p = 0.002$, cluster statistic: 4327, cluster size: 1404, largest cluster time point: 161 ms) at 130–230 ms post-stimulus latency, the difference was found in the right frontal and parietal regions. A difference was also found between the UR vs. FRE (UR > FRE, $p = 0.002$, cluster statistic: 5236, cluster size: 1706, largest cluster time point: 185 ms) at 130–230 ms post-stimulus latency and was most pronounced over the right frontal areas. No significant cluster was observed for the UR vs. PR comparison.

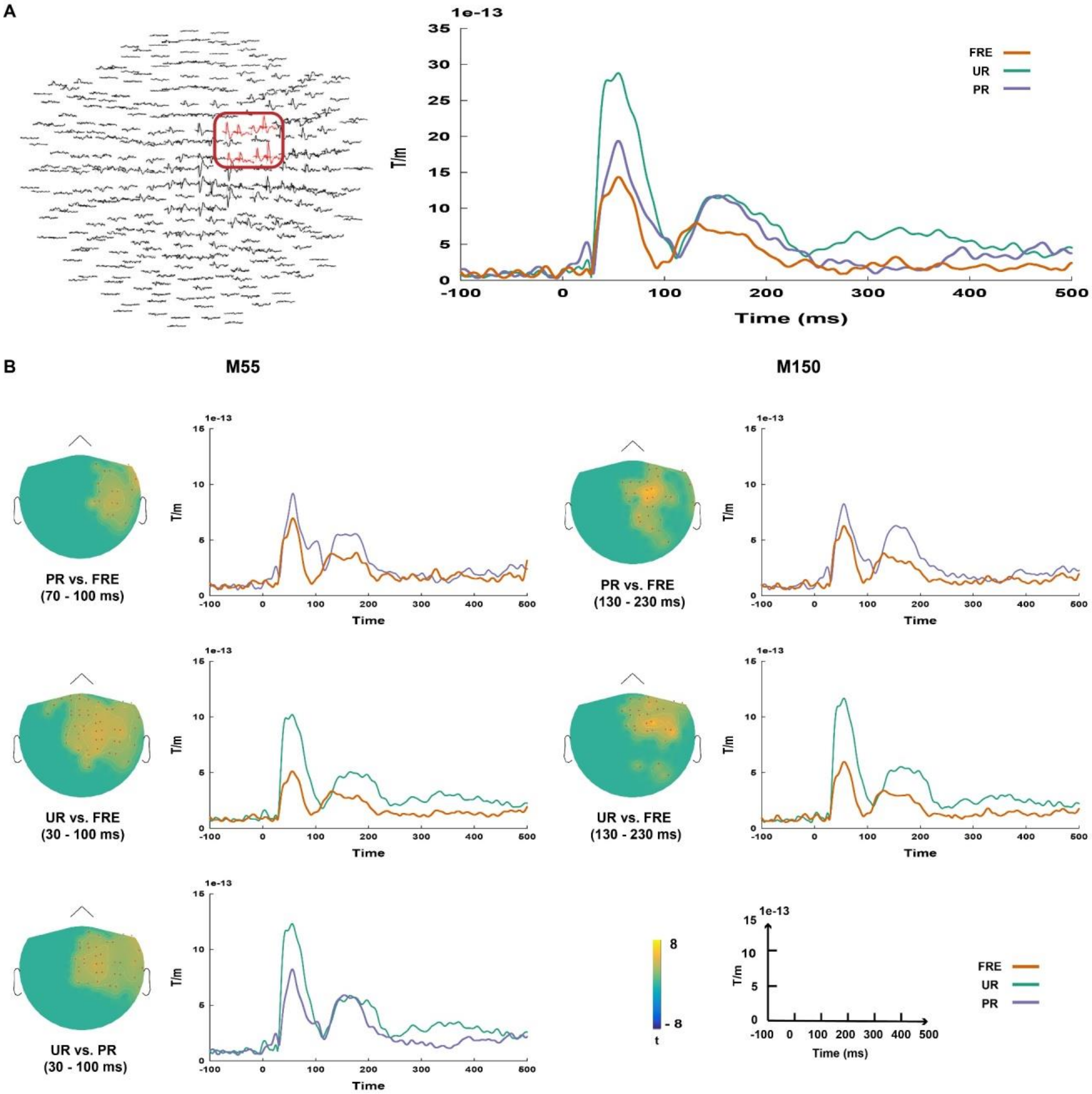

Figure 1. Sensor level results. (A) Descriptive results: *Left panel:* 306-channel sensor array viewed from the top; the grand-averaged evoked responses to the predictable rare stimuli are presented. Corresponding sensors used for the grand-averaged waveform in the right panel are marked with the red rectangle. *Right panel:* Grand-averaged evoked

responses to the UR, PR, and FRE averaged over the most activated channels. (B) Statistical analyses results. *Left panels:* significant sensor clusters of each stimulus type paired comparison shown by the red dots in the sensor space. The clusters are shown from the time point with the largest significant sensor cluster size (largest number of sensors within the cluster) in the corresponding time window below each graph. *Right panels:* the averaged sensor waveforms averaged from the significant clusters shown in the left panels. Specifically, from left to right and from top to bottom, they are: the cluster for the PR vs. FRE comparison occurring from 70 to 100 ms with the largest cluster size time point at 96 ms; the cluster for the UR vs. FRE comparison occurring from 30 to 100 ms with the largest cluster size time point at 42 ms; the cluster for the UR vs. PR comparison occurred from 30 to 100 ms, with the largest cluster size time point at 38 ms; the cluster for the PR vs. FRE comparison occurred from 130 to 230 ms, with the largest cluster size time point at 161 ms; and the cluster for the UR vs. FRE comparison occurred from 130 to 230 ms, with the largest cluster size time point at 185 ms. FRE = frequent stimulus, UR = unpredictable rare stimulus, PR = predictable rare stimulus.
